# Test–retest reliability of KINARM robot sensorimotor and cognitive assessment: in pediatric ice hockey players

**DOI:** 10.1186/s12984-015-0070-0

**Published:** 2015-09-05

**Authors:** C. Elaine Little, Carolyn Emery, Amanda Black, Stephen H. Scott, Willem Meeuwisse, Alberto Nettel-Aguirre, Brian Benson, Sean Dukelow

**Affiliations:** Department of Kinesiology, University of Calgary, Calgary, Alberta Canada; Department of Biomedical and Molecular Sciences, Queen’s University, Kingston, Ontario Canada; Departments of Pediatrics & Community Health Sciences, Alberta Children’s Hospital, University of Calgary, Calgary, Alberta Canada; Department of Clinical Neurosciences, University of Calgary, Calgary, Alberta Canada; Department of Clinical Neurosciences, Hotchkiss Brain Institute, University of Calgary, Calgary, Alberta Canada

## Abstract

**Background:**

Better diagnostic and prognostic tools are needed to address issues related to early diagnosis and management of concussion across the continuum of aging but particularly in children and adolescents. The purpose of the current study was to evaluate the reliability of robotic technology (KINARM robot) assessments of reaching, position sense, bimanual motor function, visuospatial skills, attention and decision making in youth ice hockey players (ages 10–14).

**Methods:**

Thirty-four male children attended two testing days, one week apart. On day one, each subject completed five tasks on the robot with two examiners (alternating examiner sequence); the 2^nd^ examiner followed the same procedure as the 1^st^ immediately afterwards. One consistent examiner tested subjects one week later. This is a test-retest reliability study. The robotic tasks characterize sensorimotor and/or cognitive performance; 63 parameters from 5 tasks are reported. Session 1 was the 1^st^ time the subject performed the 5 tasks, session 2 the 2^nd^ time on day 1, and session 3 one week following.

**Results:**

Intra-class correlation coefficients ranged from 0.06 to 0.91 and 0.09 to 0.90 for session 1 to 2 and 2 to 3, respectively. Bland-Altman plots showed agreement in a majority of the parameters and a learning effect in 25 % and 24 % of parameters in session 1 vs 2 and 1 vs 3, respectively but none for session 2 vs 3. Of those that showed a learning effect, only 8 % of parameters in session 1 vs 2 and 10 % in session 1 vs 3 had a clinical relevance measure ≥ 0.8.

**Conclusions:**

The relative homogeneity of the sample and the effect of learning seen in some of the task parameters appears to have negatively impacted the intra-class correlation coefficients from session 1 to 2, with less impact for 2 to 3. The Bland-Altman analysis supports good absolute reliability in healthy male children with no neurological impairment ranging in age from 10 to 14. The clinically relevant learning effect seen, in a small number of parameters could be addressed by creating a learning effect adjustment factor and/or implementing a practice session, which would eliminate the learning effect.

## Background

The incidence of concussion [or mild traumatic brain injury] in the US alone has been estimated at 1.7 million per year accounting for 80 % of all brain injuries [1–4]. One hundred and sixty thousand Canadians sustain brain injuries each year. [5]. Among Canadian university hockey players, concussion constitutes 13 % of all injuries, ranking as the second most common injury after sprains or strains [6]. More than half of mild traumatic brain injuries occur in children and adolescents [2]. Researchers from London, Ontario, Canada examined a retrospective cohort of concussions in children and adolescents (<18 years) seen in the emergency department from 2006 to 2011, and showed that of the individuals who sustained a sport-related concussion, 36 % did so while playing ice hockey [7]. Evidence suggests children and adolescents may be more susceptible to concussion, and may take longer to recover than adults [8–10]. Our understanding of the impact of sport related concussion(s) on motor and cognitive processing in children, with respect to the effect on the developing brain, is limited [11, 12].

The injury spectrum associated with concussion is broad, ranging from subtle or imperceptible to obvious changes in motor and/or cognitive performance, and vary dependent on the developmental stage of the central nervous system [13–16]. One of the primary reasons for the paucity of research related to the effect of concussion in children and adolescents is the lack of sensitive measurement tools that can identify impairments following concussion [17, 18]. Better diagnostic and prognostic tools are needed to address issues related to early diagnosis and management of concussion across the continuum of aging but particularly in children and adolescents. The scarcity of age-specific research forces practitioners to use guidelines developed for collegiate or adult populations [19]. Researchers are beginning to examine the efficacy of measurement tools used with adults among children and adolescents [20, 21]. Maturation occurs at different rates across various domains within the central nervous system, ranging broadly from 18 (reaching correction) to 30 (precision of number sense) years of age, which can complicate concussion evaluation in children and adolescents [22–24]. Clinical tools used to assess neurocognitive processing and postural control (e.g., Trail Making B Task – TMB and Balance Error Scoring System – BESS) have been evaluated to determine their reliability with children and adolescent populations [20, 21]. The BESS shows a limited ability to assess postural control in young athletes post-mild traumatic brain injury [25]. Other researchers have examined cognitive motor integration in children (mean age: 13.2 years) following concussion [26]. Subjects were required to slide a cursor from a central to a peripheral target on a dual-touchscreen laptop using one finger [26]. The results showed significant impairment in both movement timing and trajectory formation with concussion history (7 to 11 days post-concussion) [26]. Performance of the cognitive motor integration task was not restored to baseline levels until 18 months following concussion [26].

Robotic technology has the potential to offer a clinical diagnostic assessment tool that is ideal for objective, quantitative, rapid and automated assessment of neural function. The KINARM (BKIN Technologies Ltd, Ontario, Canada) is a robotic device that has been used to detect functional impairments across neurological domains [27–29]. Subjects grasp two robotic arms while performing automated upper-extremity tasks, while a two-dimensional virtual reality display serves as a visual aid in those tasks not testing proprioception. The tasks test visuomotor, proprioceptive, rapid sensorimotor and decision control, and executive function capabilities [27–29]. The KINARM end point robot has been used to explore the connection between degradation in performance on the proprioceptive task within 24 hours post mild traumatic brain injury and the prevalence of post concussion syndrome three weeks post injury [30]. Subjects in the study were > 18 years of age. The results identified subjects with post concussion syndrome had more abnormal scores than those without post concussion syndrome [30].

There is evidence that the KINARM robot is reliable and sufficiently sensitive to use in adult stroke and moderate/severe brain injury populations but little research has been published examining its reliability with children and adolescents [27–29]. Thus, the primary purpose of the current study was to evaluate the reliability of robotic technology in children and adolescents ranging in age from 10 to 14 using a series of tasks designed to assess neurological impairments. Intra-class correlation coefficients ≥ 0.50 and Bland-Altman plots associated with robotic parameters provided evidence that the KINARM robot is a reliable tool to use with children and adolescents.

## Methods

### Participants

Thirty-four healthy, normally developing boys aged 10–14 years were recruited (individuals available to attend two testing sessions one week apart) from the subject population of a 5-year longitudinal prospective study in children and adolescent ice hockey players. This was a sample of convenience. The Conjoint Health Research Ethics Board at the University of Calgary approved the study (Ethics ID number E24026). Prior to data collection, parents provided signed consent for the participants to partake in all aspects of the study and the children provided assent. Participants were included in the test-retest portion of the study if they had not previously been exposed to the robotic assessment. Individuals with prior history of concussion and no neurological signs and symptoms were included in the study. If individuals had a major injury to any joint of their upper extremities, had sustained a concussion within the month of testing and/or between test-retest sessions, or had a learning disability they were excluded from the study.

### Robotic assessment

**Fig. 1:**
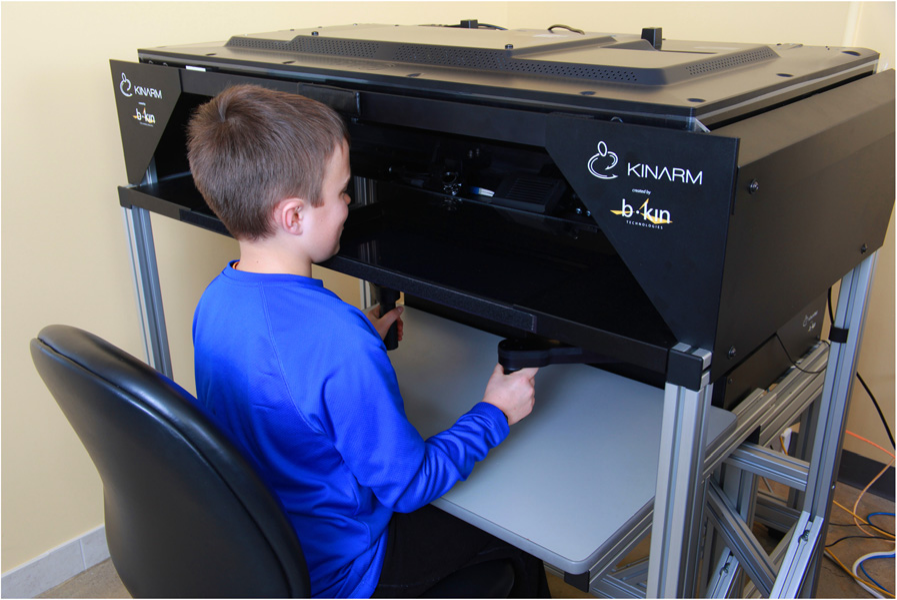
The KINARM end point robot. The virtual reality workstation makes it possible to view targets projected onto a screen

The robotic assessment was performed using the KINARM end point bimanual device, which permits free movement of the upper extremities in the horizontal plane while seated; refer to Fig. 1. A virtual reality system displays visual targets such that they appear in the same plane as the arms. Subjects experience force feedback while grasping the robot handles when hitting targets during specific tasks. Participants attended two testing sessions one week apart on the KINARM robot. On day 1, each subject completed five tasks (63 parameters total) with two examiners (alternating examiner sequence); the second examiner followed the same procedure as the first. Overall there was no reason to expect an examiner effect as all each examiner did was place the subject in front of the robot and read a predetermined set of instructions for each task. Thus the focus of the current study is the test-retest reliability for the robotic testing [31]. Each testing session lasted 17 (1) (mean (SD)) minutes and the two sessions were separated by approximately 2 minutes. Subjects were seated in a chair in front of the robot, asked to avoid slouching, and the robot height adjusted such that each child’s head rested on a location in the center of the virtual visual field. Body position was kept constant across subjects. Subjects completed the following 5 tasks during each testing session: Visually guided reaching on right and left, Arm position matching on right and left, Object hit, Object hit & avoid, and Trail making B with the dominant limb. These tasks characterize sensorimotor and/or cognitive performance. Examiner 1 from the first day of the study tested all subjects on the same five tasks (63 parameters) one week later.

### Experimental tasks

#### Visually guided reaching task

**Fig. 2:**
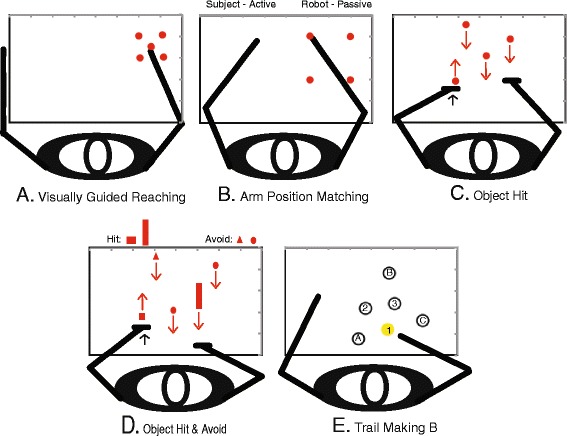
The five KINARM robot tasks used in the study. **a** Visually guided reaching with the right arm, **b** Arm position matching with the right arm, **c** Object hit, **d** Object hit and avoid, and **e**. Trail making B (not to scale, example of the alpha-numeric alternation)

This task provides a measure of upper limb visuomotor capability (Fig. 2a). The robot handle is represented as a white dot (0.5-cm radius) on the display. The task targets are red circles, each with a 1.0 cm radius. Participants reach out and back between the central and peripheral targets. The four red targets are 10 cm from the initial central target. Participants are instructed to move the white dot from the centre of one target to the centre of the next target that appears, as quickly and accurately as possible. All targets are located near the centre of the workspace for each arm. There are five blocks of trials, target location is randomized within a block and both the reach out and reach back trials are analyzed. This process is repeated forty times to explore the workspace and measure variability of the subject’s responses. Each subject completed the task twice, once with each arm; the dominant arm always preceded the non-dominant arm. Although not identical, the task used in the current work is similar to and uses metrics that were described earlier using the KINARM exoskeleton robot [28, 29, 31, 32].

#### Arm position matching task

This task provides a measure of proprioceptive (position sense) capability (Fig. 2b). The robot moves one arm (passive arm) to one of four different target locations spaced at the corners of a square grid at 20 cm intervals in the X and Y directions. Movements are made with a bell-shaped velocity profile. Then, participants actively move the opposite arm (active arm) to the mirror-image location in space. Participants notify the examiner once the mirror-matched position is reached and the examiner advances the robot to the next trial. Each participant’s vision is blocked to ensure that any sensory information about limb position comes from proprioceptive inputs. There are 6 blocks of trials, target location is randomized within a block and 1 trial for each target is completed within a block. The same target is never repeated sequentially. The task was completed twice with dominant arm being the active arm first followed by the non-dominant arm. A similar task has been used with the KINARM exoskeleton robot [28, 29, 32, 33]. To save time the task used in the current work used 4 targets rather that 8 [32].

#### Object hit task

This task is a rapid sensorimotor, decision and control test (Fig. 2c). It assesses the ability of a subject to select and engage motor actions with both hands over a range of speeds and a large workspace. Virtual paddles appear at the robot handles. Subjects are asked to use the paddles to hit virtual balls that fall from the top of the screen toward them. The robot produces a reactive force that mimics the actual force that would have been felt by the subject if these were real objects contacting a real paddle. As the task proceeds the balls move at greater speeds and appear more often, making the task more difficult as time progresses. Balls fall at random from ten bins, which are spread equally across the workspace, and thirty balls fall from each bin. A total of three hundred balls are dropped during the task in one minute and forty-four seconds. A similar task has been used with older adults and the KINARM exoskeleton robot, with a slight reduction in the total time the balls were dropped for the current work [34].

#### Object hit and avoid

This task is similar to the Object hit task, but requires higher executive function. Participants must hit target objects while avoiding all others (Fig. 2d). Thus the emphasis is on attention, rapid motor selection, and inhibition. At the start of the task subjects are shown two target shapes of a possible eight, they are instructed to memorize these as the only two shapes to hit during the task, and to avoid all other (6) distractor shapes. If distractors hit the participant’s paddles they pass through the paddles but there is no reactive force felt by the subject. This provides immediate and ongoing feedback to the subject that the object was a distracter and not a target. As with the preceding task, when targets are hit the robot produces a reactive force that mimics the actual force that would have been felt by the subject if these were real objects contacting a real paddle. Two hundred objects and one hundred distractors fall in just over [Bourke TC, Lowrey CR, Dukelow SP, Bagg SD, Norman KE, Scott SH. A robot-based behavioural task to quantify impairments in rapid motor decisions and actions after stroke. Submitted].

#### Trail making B task

This task is the second part of a cognitive test that evaluates executive function (e.g., visual attention and task switching) from the field of neuropsychology that is commonly used in the assessment of brain injury (Fig. 2e) [35]. Normative data from pen and pencil versions of the task have been published for adolescents, adults and older adults across age ranges of 15–20, 20–59, and 55–85, respectively. [36]. Participants trace through an alternating alpha-numeric sequence of targets 1-A-2-B for example, up to 13, for a total of 25 targets. A shortened version of the task that has 5 targets precedes the full task to help familiarize subjects with the task. If the subject touches an incorrect target while moving through the sequence the preceding correct target will turn red and the subject must return to that target before continuing. There are eight possible patterns for the Trail making B task [37]. These patterns were randomly presented within and across subjects who participated in the study.

#### Outcome measures

Task parameters associated with each task are presented in Table 1. The parameters for each task were developed to quantify task performance, thus behavioral attributes associated with the parameters are included in Table 1.

**Table 1:** A summary of the five KINARM robot tasks

Task	Behavioral attribute	Parameter	Definition
Visually Guided Reaching R & L	Upper Limb Postural Control	Posture speed (m/s)	Mean hand speed when the hand should be at rest.
	Motor Response to a Visual Stimulus	Reaction time (s)	Time from target onset to movement onset.
	Feed-forward Control: Initial phase of the movement.	Initial direction error (rad)	Angular deviation between (i) a straight line from the hand position at movement onset to the destination target, (ii) a straight line from the hand position at movement onset to the hand position after the initial phase of movement.
	Feed-forward Control: Initial phase of the movement.	Initial distance ratio	Ratio of (i) the distance the hand travelled during the subject’s initial phase of movement to (ii) the distance the hand travelled between movement onset and movement offset (or the end of the trial if the destination target is not reached).
	Feed-forward Control: Initial phase of the movement.	Initial speed ratio	Ratio of (i) the maximum hand speed during the subject’s initial phase of movement to (ii) the global hand speed maximum of the trial.
	Feedback Control: Movement corrections after the initial motor response.	Speed maxima count	Number of hand speed maxima between movement onset and offset.
	Feedback Control: Movement corrections after the initial motor response.	Minimum maximum speed difference (m/s)	Differences between hand speed maxima and minima.
	Total Movement	Movement time (s)	Total time elapsed from movement onset to end.
	Total Movement	Path length ratio	Ratio of (i) the distance travelled by the hand between the movement onset and movement offset and (i) the straight line distance between the starting and destination targets.
	Total Movement	Max speed (m/s)	Global maximum hand speed.
Arm position matching R & L	Position Sense	Variability XY (m)	Root-mean-square (RMS) of X and Y variables: mean value of the variability of the subject’s hand position in the X and Y directions.
	Position Sense	Contraction/expansion ratio XY	Ratio of the range of area moved over – arm moved by the subject compared to the arm moved by the robot. Ratio of range of movement in the x and y directions are used in the current ratio.
	Position Sense	Shift XY (m)	RMS of the X and Y shifts: mean difference between the mirrored x and y positions of the arm moved by the subject and the x and y positions of the arm (+ lateral shift, - median shift).
	Position Sense	Absolute Error XY	The mean absolute distance error across all trials.
Object Hit	Global Performance	Total hits	Number of balls hit off the screen in the opposite direction from it original path.
	Global Performance	Hits with left	Number of balls hit with the left (L) hand
	Global Performance	Hit with right	Number of balls hit with the right (R) hand
	Motor Performance	Hand bias hits	Quantifies which hand is used more often for hitting the balls (hand dominance).
	Spatial & Temporal Performance	Miss bias	Quantifies any bias of misses toward one side of the work space or the other (x direction only).
	Spatial & Temporal Performance	Hand transition	Shows where the subject’s preference for using one hand over the other switches in the work space.
	Motor Performance	Hand selection overlap	Captures how effective subjects are at using both hands and how often they overlap hands (i.e., hit balls with both the R and L hands in the same area of the work space).
	Spatial & Temporal Performance	Median error	The percentage of the way through the task when the subject made half their errors.
	Motor Performance	Hand speed L (m/s)	The mean L hand speed maintained through the entire task.
	Motor Performance	Hand speed R (m/s)	The mean R hand speed maintained through the entire task.
		Hand bias	Value from -1 to 1 that describes the bias in hand speed between the hands.
	Motor Performance	Movement area L & R (m^2)	Area of space the subject used with each hand during the task.
	Motor Performance	Movement area bias	Value from -1 to 1 that describes the bias in movement area between hands.
Object Hit & Avoid	Includes the 14 parameters from Object Hit	Distractor hits L	Number of distractor objects hit with L hand.
	Global Performance	Distractor hits R	Number of distractor objects hit with the R hand.
	Global Performance	Distractor hits total	Number of distractor objects the subject hit; reported as the % of total distracters dropped.
Trail Making B	Executive Function	Total time (s)	Total time from the targets being illuminated to touching the last target.
	Executive Function	Dwell time (s)	Total time spent with the hand feedback dot at the targets.
		Time ratio	Time for targets 13/25/time for targets 1–12.
		Error count	Number of times an incorrect target was touched.

**Legend:** Behavioral attributes and definitions of task parameters are outlined

#### Data analysis

Statistical analyses were performed in SPSS, version 19.0 [38]. The study was a repeated-measures design. Significance level was set at alpha = 0.05. All subjects and their data were included in the analysis as there were no missing data points. In general, an effect size of 0.10 was considered small, 0.30 moderate, and 0.50 large [38]. The effect size is expressed as focused comparisons based on any interactions or main effects identified [38]. Data analysis was based on session; session one (S1) refers to the first time the subject performed the 5 tasks and session two (S2) the second time the five tasks were performed all on day one. Session 3 (S3) was performed following one week.

Intra-class correlation coefficients were used to assess consistency or reliability of outcomes from the KINARM robot for S1 to S2 and S2 to S3 [38, 39]. Although there are no standard values for acceptable relative reliability associated with intra-class correlation coefficients, the following general guidelines have been suggested, values > 0.75 indicate good reliability and < 0.75 poor to moderate reliability [31]. Researchers and clinicians have been encouraged not to use these general guidelines as absolute standards but to remember that the degree of acceptable precision in the measurement must be taken into account when determining an acceptable reliability cut-off point [31]. For the purposes of the current study coefficients of < 0.50 will indicate poor reliability, coefficients from 0.50 to < 0.75 moderate reliability, and coefficients ≥ 0.75 good reliability [31]. In the current study the intra-class correlation model was a two-way repeated measures, random effects analysis of variance (ANOVA) model and type consistency was performed using SPSS. Session was used as the random sample to compute the intra-class correlation coefficients [38–40].

Bland-Altman plots were used to evaluate agreement for S1 to S2 and S2 to S3 and reflect the spread of difference scores (e.g., S1 – S2) around the line of equality, the line all points would lie on if outcomes were exactly the same when tested across sessions, the line at zero on the graph [31, 41–42]. The spread of the difference scores indicates whether the level of observed error is acceptable, in the current study when S1 is substituted for S2 and S2 for S3 [31, 42, 43]. The sessions are considered to be in agreement when the difference in subject’s performance for S1 to S2 or S2 to S3 is small enough, within an acceptable clinical error range, for the methods to be considered interchangeable [31, 41–43]. The 95 % limits of agreement define the range within which most differences between measurements will lie based on difference scores for S1 to S2 and S2 to S3. The requirements for agreement are met when 95 % of these difference scores fall within two standard deviations above and below the mean of the difference scores [31, 41, 42].

Two-way repeated measures ANOVAs (parameters by sessions) were used to identify interaction and/or session effects for S1 to S2, S2 to S3, and S1 to S3 for right and left hands with the Visually guided reaching and Arm position matching tasks, as well as for the Object hit, Object hit and avoid, and Trail making B tasks. Post-hoc Bonferroni corrections were used to determine those parameters that showed a significant learning effect with improvement in the presence of a session effect. Only those parameters showing significant improvement in performance were analyzed to determine clinical relevance based on the individual effect size standards measure [43, 44]. This will be referred to as the clinical relevance measure in the current study and was determined using the following formula:

$$ {\partial}_{\mathrm{group}} = {\mathrm{m}}_2\hbox{-}\ {\mathrm{m}}_1/{\mathrm{s}}_1 $$

where

∂ _group_ = clinical relevance measure for the group

m_1_ = the group mean at baseline

m_2_ = the group mean at follow-up

s_1_ = the group standard deviation at baseline [43–45].

Group effect size standards for the clinical relevance measure are 0.20 for a small group change, 0.50 for a moderate group change, and 0.80 for a large group change [45, 46]. The cut-off benchmark of ≥ 0.8 was selected to coincide with clinical relevance in the current paper [45, 46].

## Results

Characteristics of the subjects who took part in the study can be found in Table 2. Table 3 presents a summary of the intra-class correlation coefficients and the associated 95 % confidence intervals for parameters bilaterally for Visually guided reach, Arm position matching, and then for Object hit, Object hit and avoid, and the Trail making B tasks. Intra-class correlation coefficients were <0.50 in 25 %, ≥ 0.50 to < 0.75 in 49 %, and ≥ 0.75 in 26 % of the parameters for S1 to S2 and < 0.50 in 27 %, ≥ 0.50 to < 0.75 in 37 %, and ≥ 0.75 in 36 % of the parameters for S2 to S3.

**Table 2:** Summary of the study population characteristics

Number of subjects	34
Age	11.5(1.1)
Range	10–14 years
Height (cm)	154.6 ± 9.6
Weight (Kg)	43.3 ± 9.6
Gender	M =34
Dominant Hand	R = 31
	L = 3
History of Concussion	0 = 25
	1 = 6
	2 = 3

**Table 3:** Summary of Intra-class correlation coefficients and 95 % Confidence intervals

Visually Guided Reaching Parameters: R	ICC: S1 vs S2	ICC: S2 vs S3
Posture speed (m/s)	0.65, CI (0.30, 0.83)	0.38, CI (-0.24, 0.69)
Reaction time (s)	0.91, CI (0.81, 0.95)	0.84, CI (0.68, 0.92)
Initial direction error (rad)	0.48, CI (-0.05, 0.74)	0.72, CI (0.44, 0.86)
Initial distance ratio	0.12, CI (-0.76, 0.56)	-0.10, CI (-1.21, 0.45)
Initial speed ratio	0.11, CI (-0.79, 0.55)	0.43, CI (-0.15, 0.72)
Speed maxima count	0.61, CI (0.21, 0.80)	0.40, CI (-0.19, 0.70)
Minimum maximum speed difference (m/s)	0.73, CI (0.47, 0.87)	0.73, CI (0.47, 0.87)
Movement time (s)	0.61, CI (0.22, 0.80)	0.76, CI (0.53, 0.88)
Path length ratio	0.76, CI (0.52, 0.88)	0.77, CI (0.55, 0.89)
Max speed (m/s)	0.76, CI (0.53, 0.88)	0.83, CI (0.66, 0.92)
Visually Guided Reaching Parameters: L		
Posture speed (m/s)	0.74, CI (0.48, 0.87)	0.76, CI (0.52, 0.88)
Reaction time (s)	0.89, CI (0.78, 0.95)	0.80, CI (0.51, 0.90)
Initial direction error (rad)	0.73, CI (0.47, 0.87)	0.75, CI (0.50, 0.87)
Initial distance ratio	0.67, CI (0.33, 0.83)	0.71, CI (0.41, 0.85)
Initial speed ratio	0.30, CI (-0.40, 0.65)	0.74, CI (0.48, 0.87)
Speed maxima count	0.73, CI (0.46, 0.87)	0.69, CI (0.39, 0.85)
Minimum maximum speed difference (m/s)	0.81, CI (0.61, 0.90)	0.80, CI (0.60, 0.90)
Movement time (s)	0.75, CI (0.49, 0.87)	0.88, CI (0.76, 0.94)
Path length ratio	0.90, CI (0.80, 0.95)	0.90, CI (0.79, 0.95)
Max speed (m/s)	0.79, CI (0.59, 0.897)	0.90, CI (0.79, 0.95)
Arm Position Matching Parameters: R		
Variability XY (m)	0.29, CI (-0.41, 0.65)	0.38, CI (-0.24, 0.69)
Contraction/expansion ratio XY	0.86, CI (0.72, 0.93)	0.87, CI (0.74, 0.93)
Shift XY (m)	0.59, CI (0.18, 0.80)	0.27, CI (-0.47, 0.63)
Absolute Error XY	0.66, CI (0.32, 0.83)	0.54, CI (0.07, 0.77)
Arm Position Matching Parameters: L		
Variability XY (m)	0.62, CI (0.24, 0.81)	0.51, CI (0.01, 0.75)
Contraction/expansion ratio XY	0.77, CI (0.54, 0.88)	0.77, CI (0.54, 0.89)
Shift XY (m)	0.67, CI (0.34, 0.84)	0.43, CI (-0.14, 0.72)
Absolute Error XY	0.83, CI (0.65, 0.91)	0.51, CI (0.03, 0.76)
Object Hit Parameters	ICC: S1 vs S2	ICC: S2 vs S3
Total hits	0.79, CI (0.58, 0.90)	0.88, CI (0.76, 0.94)
Hits with left	0.75, CI (0.51, 0.88)	0.83, CI (0.64, 0.92)
Hit with right	0.74, CI (0.49, 0.87)	0.85, CI (0.70, 0.93)
Hand bias hits	0.60, CI (0.21, 0.80)	0.73, CI (0.45, 0.86)
Miss bias	0.67, CI (0.33, 0.83)	0.89, CI (0.78, 0.95)
Hand transition	0.62, CI (0.24, 0.81)	0.71, CI (0.43, 0.86)
Hand selection overlap	0.43, CI (-0.15, 0.71)	0.23, CI (-0.54, 0.62)
Median error	0.30, CI (-0.40, 0.65)	0.42, CI (-0.17, 0.71)
Hand speed L (m/s)	0.76, CI (0.53, 0.88)	0.79, CI (0.59, 0.90)
Hand speed R (m/s)	0.80, CI (0.59, 0.90)	0.79, CI (0.58, 0.89)
Hand speed bias	0.72, CI (0.43, 0.86)	0.75, CI (0.50, 0.88)
Movement area L (m^2)	0.56, CI (0.12, 0.78)	0.50, CI (0.00, 0.75)
Movement area R (m^2)	0.65, CI (0.31, 0.83)	0.83, CI (0.67, 0.92)
Movement area bias	0.64, CI (0.27, 0.82)	0.40, CI (-0.21, 0.70)
Object Hit & Avoid Parameters		
Total hits	0.71, CI (0.41, 0.85)	0.65, CI (0.29, 0.82)
Hits with left	0.83, CI (0.65, 0.91)	0.53, CI (0.06, 0.77)
Hit with right	0.43, CI (-0.14, 0.72)	0.55, CI (0.09, 0.77)
Hand bias hits	0.48, CI (-0.04, 0.74)	0.44, CI (-0.29, 0.68)
Miss bias	0.69, CI (0.38, 0.85)	0.60, CI (0.19, 0.80)
Hand transition	0.58, CI (0.15, 0.79)	0.48, CI (-0.04, 0.74)
Hand selection overlap	0.36, CI (-0.28, 0.68)	0.63, CI (0.27, 0.82)
Median error	0.12, CI (-0.77, 0.56)	0.49, CI (-0.03, 0.74)
Hand speed L (m/s)	0.75, CI (0.49, 0.87)	0.57, CI (0.13, 0.78)
Hand speed R (m/s)	0.78, CI (0.56, 0.89)	0.76, CI (0.52, 0.88)
Hand speed bias	0.69, CI (0.37, 0.84)	0.64, CI (0.27, 0.82)
Movement area L (m^2)	0.53, CI (0.06, 0.77)	0.47, CI (-0.07, 0.73)
Movement area R (m^2)	0.70, CI (0.40, 0.85)	0.67, CI (0.34, 0.84)
Movement area bias	0.00, CI (-0.99, 0.50)	0.32 CI (-0.37, 0.66)
Distractor hits L	0.42, CI (-0.16, 0.71)	0.72, CI (0.44, 0.86)
Distractor hits R	0.60, CI (0.20, 0.80)	0.84, CI (0.68, 0.92)
Distractor hits total	0.54, CI (0.09, 0.72)	0.85, CI (0.70, 0.93)
Trail Making B Parameters		
Total time (s)	0.44, CI (-0.13, 0.72)	0.67, CI (0.35, 0.84)
Dwell time (s)	0.71, CI (0.42, 0.86)	0.72, CI (0.43, 0.86)
Time ratio	0.06, CI (-0.89, 0.53)	-0.35, CI (-1.70, 0.33)
Error count	0.10, CI (-0.80, 0.55)	0.09, CI (-0.82, 0.55)

**Legend:** Statistics from S1 to S2 and S2 to S3 in parameters associated with Visually guided reach, Arm position matching, Object hit, Object hit and avoid, and Trail making B tasks

**Fig. 3:**
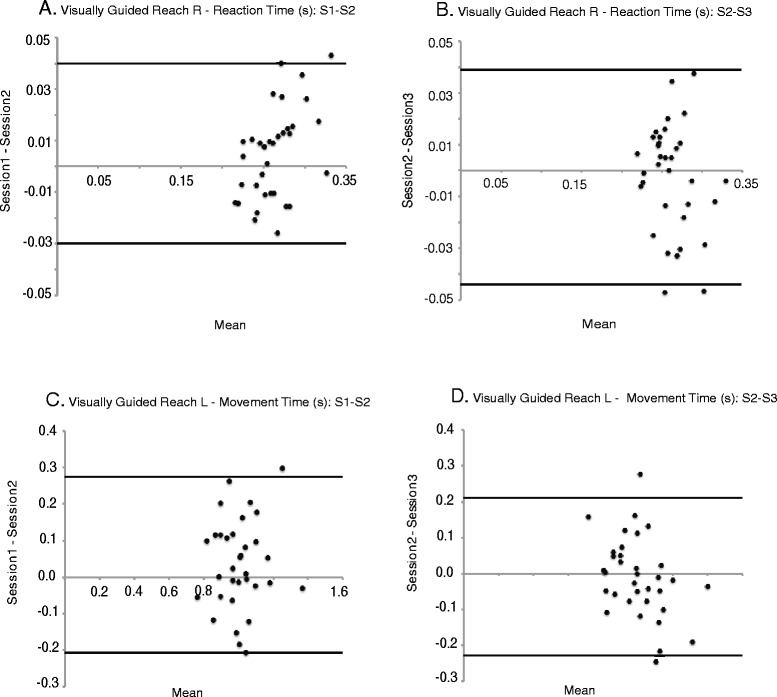
Bland-Altman plots. **a** and **b**: Reaction time (s) for S1 to S2 and S2 to S3, respectively. **c** and **d**: Movement time (s) for S1 to S2 and S2 to S3, respectively. The difference scores fall primarily within the 95 % upper and lower limits of agreement

**Fig. 4:**
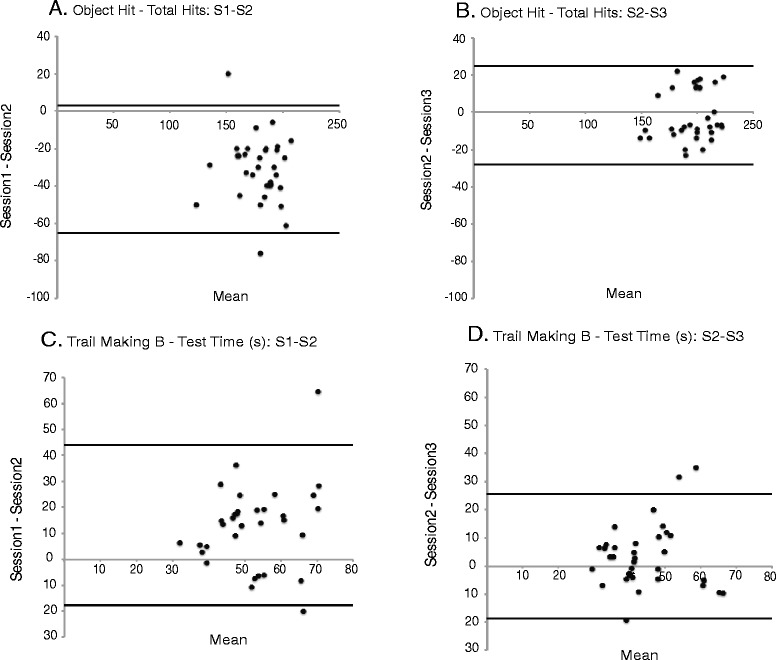
Bland-Altman plots. **a** and **b**: Total hits for S1 to S2 and S2 to S3, respectively. **a** shows a learning effect with a negative shift in the difference scores. **c** and **d**: Test time (s) for S1 to S2 and S2 to S3, respectively. **c** shows a learning effect with a positive shift in the difference scores while **b** and **d** reflect the maintenance of the learning effect one week following the initial testing session

Table 4 includes a summary of the data used when determining agreement related to Bland-Altman plots, which are presented in Figs. 3 and 4. The Bland-Altman plots suggest agreement in the majority of the parameters across the five tasks evaluated however a few parameters showed a learning effect. Figure 3 presents Bland-Altman plots comparing S1 to S2 and S2 to S3 for both reaction time (s) (Visually guided reach-R) and movement time (s) (Visually guided reach-L). All errors appear unbiased as differences are spread evenly and randomly above and below the line of equality in Fig. 3a, b, c, and d. Alternatively, Fig. 4a shows a negative shift in the difference scores related to total hits (Object hit) for S1 to S2 which reflects the presence of a learning effect. When S2 was compared to S3, the learning effect appears to be have been maintained over one week when the subject returned to repeat the testing (Fig. 4b). Figure 4c and d represent test time (s) (Trail making B) for S1 to S2 and S2 to S3, respectively. Although the shift in difference scores seen in Fig. 4c is in the positive direction this also reflects a learning effect. As seen with the previous parameter Fig. 4d shows that the learning effect was maintained over the one week when the subject returned to repeat the testing procedure.

**Table 4:** Summary data associated with Bland-Altman plots

Visually Guided Reaching Parameters: R	Mean Difference (SD)	95 % Limits of Agreement	Mean Difference (SD)	95 % Limits of Agreement
	S1 to S2	S1 to S2	S2 to S3	S2 to S3
Posture speed (m/s)	-0.0001 (0.001)	(-0.003, 0.002)	0.0005 (0.002)	(-0.003, 0.004)
Reaction time (s)	0.005 (0.018)	(-0.030, 0.040)	-0.002 (0.021)	(-0.044, 0.039)
Initial direction error (rad)	0.006 (0.012)	(-0.019, 0.031)	-0.001 (0.010)	(-0.020, 0.019)
Initial distance ratio	-0.027 (0.055)	(-0.137, 0.083)	0.010 (0.070)	(-0.130, 0.151)
Initial speed ratio	-0.002 (0.028)	(-0.057, 0.054)	-0.004 (0.023)	(-0.049, 0.041)
Speed maxima count	0.111 (0.415)	(-0.718, 0.942)	-0.015 (0.495)	(-1.006, 0.976)
Minimum maximum speed difference (m/s)	0.006 (0.006)	(-0.006, 0.018)	-0.003 (0.008)	(-0.019, 0.012)
Movement time (s)	0.023 (0.149)	(-0.274, 0.320)	0.112 (0.415)	(-0.718, 0.942)
Path length ratio	0.045 (0.050)	(-0.055, 0.145)	-0.014 (0.055)	(-0.124, 0.096)
Max speed (m/s)	0.007 (0.058)	(-0.110, 0.123)	-0.005 (0.068)	(-0.140, 0.131)
Visually Guided Reaching Parameters: L				
Posture speed (m/s)	-0.0003 (0.001)	(-0.003, 0.002)	-0.0002 (0.002)	(-0.003, 0.003)
Reaction time (s)	0.010 (0.018)	(-0.046, 0.026)	0.0001 (0.027)	(-0.054, 0.054)
Initial direction error (rad)	0.006 (0.013)	(-0.020, 0.031)	-0.001 (0.014)	(-0.029, 0.027)
Initial distance ratio	-0.010 (0.042)	(-0.094, 0.074)	0.004 (0.045)	(-0.085, 0.094)
Initial speed ratio	0.010 (0.032)	(-0.054, 0.073)	-0.002 (0.027)	(-0.057, 0.053)
Speed maxima count	0.099 (0.313)	(-0.527, 0.725)	-0.027 (0.342)	(-0.711, 0.658)
Minimum maximum speed difference (m/s)	0.002 (0.008)	(-0.013, 0.018)	0.0007 (0.008)	(-0.015, 0.017)
Movement time (s)	0.034 (0.120)	(-0.207, 0.274)	-0.009 (0.110)	(-0.228, 0.210)
Path length ratio	0.037 (0.047)	(-0.057, 0.132)	0.003 (0.052)	(-0.102, 0.108)
Max speed (m/s)	0.003 (0.062)	(-0.121, 0.128)	-0.009 (0.060)	(-0.128, 0.111)
Arm Position Matching Parameters: R				
Variability XY (m)	0.001 (0.012)	(-0.022, 0.024)	0.001 (0.012)	(-0.022, 0.024)
Contraction/expansion ratio XY	-0.026 (0.155)	(-0.335, 0.283)	-0.051 (0.157)	(-0.366, 0.263)
Shift XY (m)	0.004 (0.029)	(-0.054, 0.062)	-0.005 (0.031)	(-0.066, 0.057)
Absolute Error XY	0.002 (0.022)	(-0.041, 0.046)	-0.001 (0.022)	(-0.044, 0.042)
Arm Position Matching Parameters: L				
Variability XY (m)	0.003 (0.012)	(-0.021, 0.027)	0.001 (0.011)	(-0.022, 0.023)
Contraction/expansion ratio XY	-0.001 (0.168)	(-0.338, 0.336)	-0.020 (0.175)	(-0.371, 0.330)
Shift XY (m)	-0.008 (0.023)	(-0.055, 0.038)	0.004 (0.037)	(-0.051, 0.059)
Absolute Error XY	-0.005 (0.015)	(-0.034, 0.025)	0.003 (0.021)	(-0.039, 0.045)
Object Hit Parameters				
Total hits	-31 (17)	(-65, 3)	(-2 (13))	(-28, 25)
Hits with left	-12 (10)	(-33, 8)	-3 (9)	(-21, 15)
Hit with right	-19 (11)	(-41, 4)	1 (9)	(-17, 19)
Hand bias hits	-0.024 (0.077)	(-0.179, 0.131)	0.024 (0.064)	(-0.104, 0.153)
Miss bias	0.008 (0.035)	(-0.063, 0.078)	-0.009 (0.025)	(-0.059, 0.041)
Hand transition	-0.002 (0.032)	(-0.066, 0.062)	-0.005 (0.028)	(-0.061, 0.051)
Hand selection overlap	-0.010 (0.048)	(-0.106, 0.086)	-0.013 (0.048)	(-0.109, 0.082)
Median error	-5 (4)	(-12, 3)	-0.941 (4)	(-9, 8)
Hand speed L (m/s)	-0.038 (0.052)	(-0.142, 0.065)	-0.003 (0.046)	(-0.095, 0.090)
Hand speed R (m/s)	-0.041 (0.055)	(-0.151, 0.070)	0.011 (0.055)	(-0.098, 0.120)
Hand speed bias	-0.0004 (0.060)	(-0.056, 0.060)	0.022 (0.058)	(-0.095, 0.138)
Movement area L (m^2)	-0.020 (0.027)	(-0.075, 0.035)	-0.001 (0.028)	(-0.058, 0.055)
Movement area R (m^2)	-0.021 (0.028)	(-0.077, 0.036)	-0.001 (0.021)	(-0.042, 0.041)
Movement area bias	0.004 (0.076)	(-0.149, 0.156)	-0.0004 (0.091)	(-0.183, 0.182)
Object Hit & Avoid Parameters				
Total hits	-11 (13)	(-37, 16)	-1 (13)	(-27, 26)
Hits with left	-6 (6)	(-18, 6)	-1 (9)	(-18, 17)
Hit with right	-5 (11)	(-26, 17)	0.1 (10)	(-20, 20)
Hand bias hits	0.017 (0.094)	(-0.170, 0.204)	0.011 (0.110)	(-0.209, 0.230)
Miss bias	0.006 (0.036)	(-0.077, 0.066)	0.004 (0.041)	(-0.078, 0.085)
Hand transition	-0.003 (0.034)	(-0.071, 0.065)	-0.004 (0.041)	(-0.086, 0.077)
Hand selection overlap	-0.019 (0.045)	(-0.109, 0.071)	0.004 (0.037)	(-0.069, 0.078)
Median error	-1 (6)	(-13, 11)	-1 (5)	(-11, 9)
Hand speed L (m/s)	-0.027 (0.032)	(-0.092, 0.038)	0.001 (0.044)	(-0.087, 0.089)
Hand speed R (m/s)	-0.019 (0.044)	(-0.107, 0.070)	0.007 (0.047)	(-0.086, 0.100)
Hand speed bias	0.024 (0.079)	(-0.133, 0.182)	0.007 (0.091)	(-0.175, 0.190)
Movement area L (m^2)	-0.017 (0.024)	(-0.065, 0.030)	0.0004 (0.028)	(-0.057, 0.058)
Movement area R (m^2)	-0.011 (0.023)	(-0.057, 0.035)	0.0004 (0.023)	(-0.046, 0.047)
Movement area bias	0.025 (0.117)	(-0.210, 0.260)	-0.003 (0.121)	(-0.244, 0.238)
Distractor hits L	-1 (4)	(-10, 8)	0 (4)	(-8, 9)
Distractor hits R	-2 (5)	(-11, 8)	1 (4)	(-6, 8)
Distractor hits total	-3 (8)	(-20, 13)	1 (6)	(-11, 13)
Trail Making B Parameters				
Total time (s)	13 (15)	(-18, 44)	3 (11)	(-19, 26)
Dwell time (s)	6 (7)	(-8, 20)	3 (7)	(-10, 16)
Time ratio	-0.054 (0.424)	(-0.902, 0.795)	-0.045 (0.550)	(-1.145, 1.055)
Error count	1 (3)	(-5, 6)	0 (2)	(-4, 5)

**Legend:** Mean Difference and 95 % Limits of Agreement (lower followed by upper limit) for S1 to S2 and S2 to S3 for parameters associated with Visually guided reach, Arm position matching, Object hit, Object hit and avoid, and Trail making B tasks

Outcomes from the two-way repeated measures ANOVAs (parameters by sessions) are presented in Table 5. Interactions between parameters and sessions were identified for Object hit, Object hit and avoid, and Trail making B tasks with Bonferroni adjustment showing a main effect of session for S1 to S2 and S1 to S3 in each but not for S2 to S3. This further supports the evidence seen in the Bland-Altman plots that the learning effect had stabilized after the second completion of the tasks, as no significant improvement in performance was seen from S2 to S3. Table 5 also includes *p*-values from post hoc Bonferroni corrections for a few parameters that indicate the presence of a learning effect with the Object hit, Object hit and avoid, and Trail making B tasks for S1 to S2 and S1 to S3. The clinical relevance measure reflects a clinically relevant change between sessions and coincides with a value of ≥ 0.80.

**Table 5:** Outcomes from the two-way repeated measures ANOVAs (parameters by sessions)

KINARM Robot Tasks	Visually Guided Reach R	Visually Guided Reach L	Arm Position Matching R	Arm Position Matching L	Object Hit	Clinical Relevance	Object Hit and Avoid	Clinical Relevance	Trail Making B	Clinical Relevance
Two-way repeated measures ANOVA (parameters by session)	No interaction: F(18, 16) = 2.062, *p* < 0.076	Interaction: F(18, 16) = 9.888, *p* < 0.0001, r = 0.4	No interaction: F(6, 28) = 2.299, *p* < 0.063	No interaction:F(6, 28) = 1.137, *p* < 0.367	Interaction: F(25, 9) = 12.411, *p* < 0.0001, r = 0.8	NA	Interaction: F(30, 4) = 5.896, *p* < 0.048, r = 0.8	NA	Interaction: F(6, 28) =11.566, *p* < 0.0001, r = 0.5	NA
Bonferroni Adjustment for the main effect of Session.	S1vsS2: *p* < 0.134	S1vsS2 *p* < 0.069	S1vsS2 *p* < 1.000	S1vsS2 *p* < 1.000	***S1vsS2 p < 0.0001***	NA	***S1vsS2 p < 0.001***	NA	***S1vsS2 p < 0001***	NA
	S2vsS3 *p* < 1.000	S2vsS3 *p* < 1.000	S2vsS3 *p* < 0.163	S2vsS3 *p* < 1.000	S2vsS3 *p* < 1.000		S2vsS3 *p* < 1.000		S2vsS3 *p* < 0.07	
	S1vsS3 *p* < 0.811	S1vsS3 *p* < 0.343	S1vsS3 *p* < 0.068	S1vsS3 *p* < 1.000	***S1vsS3 p < 0.0001***		***S1vsS3 p < 0.0001***		***S1vsS3 p < 0.0001***	
Post-hoc Bonferroni corrections	NA	NA	NA	NA	1) Total hits: *p* < 0.0001	**1.58**	1) Total hits: *p* < 0.0001	0.70	1) Test time (s): *p* < 0.001	**0.92**
S1 vs S2										
					2) Hits with left: *p* < 0.0001	**1.10**	2) Hits with left: *p* < 0.0001	0.73	2) Dwell time (s): *p* < 0.0001	0.78
					3) Hits with right: *p* < 0.0001	**1.52**	3) Hits with right: *p* < 0.020	0.47		
					4) Hand speed left (m/s): *p* < 0.0001	0.62	4) Hand Selection Overlap: *p* < 0.021	0.49		
					5) Hand speed right (m/s): *p* < 0.0001	0.57	5) Hand speed left (m/s): *p* < 0.0001	0.63		
					6) Movement area left hand (m^2): *p* < 0.0001	0.71	6) Hand speed right (m/s): *p* < 0.021	0.35		
					7) Movement area right hand (m^2): *p* < 0.0001	0.73	7) Movement area left hand (m^2): *p* < 0.0001	**0.80**		
							8) Movement area right hand (m^2): *p* < 0.009	0.43		
Post-hoc Bonferroni corrections:	NA	NA	NA	NA	1) Total hits: *p* < 0.0001	**1.66**	1) Total hits: *p* < 0.0001	0.75	1) Test time (s): *p* < 0.0001	**1.16**
S1 to S3										
					2) Hits with left: *p* < 0.0001	**1.38**	2) Hits with left: *p* < 0.0001	**0.84**	2) Dwell time (s): *p* < 0.0001	**1.13**
					3) Hits with right: *p* < 0.0001	**1.40**	3) Hits with right: *p* < 0.014	0.46		
					4) Hand Selection Overlap: *p* < 0.021	0.62	4) Hand speed left (m/s): *p* < 0.002	0.62		
					5) Hand speed left (m/s): *p* < 0.0001	0.67	5) Movement area left hand (m^2): *p* < 0.001	0.78		
					6) Hand speed right (m/s): *p* < 0.025	0.42	6) Movement area right hand (m^2): *p* < 0.032	0.41		
					7) Movement area left hand (m^2): *p* < 0.0001	0.75				
					8) Movement area right hand (m^2): *p* < 0.0001	0.76				

**Legend:** Statistical analysis summary that included interactions (bold italic) and session effects across the five KINARM tasks as well as identification of those parameters that showed a statistically significant improvement in performance from session 1 to 2 and session 1 to 3. A clinically relevant change coincides with a value of ≥ 0.8 (presented in bold)

## Discussion

The main purpose of the current study was to evaluate the reliability of the KINARM robot with the objective of using it as an assessment tool to evaluate motor and/or cognitive performance in male children and adolescents ranging in age from 10 to 14. One of the strengths of the current study is that performance outcome reliability was evaluated using relative reliability (intra-class correlation coefficients ≥ 0.50) and absolute reliability (Bland-Altman agreement) methodologies.

Intra-class correlation coefficients tended to be moderate to high for most parameters across tasks and sessions. Intra-class correlation coefficients have been computed previously for the KINARM robot tasks of Visually guided reaching and Arm position matching in two separate populations that included both young adults and older adults who had suffered a stroke [27, 32]. In the study that evaluated Visually guided reaching, 25 % of parameters were ≥ 0.50 to < 0.75 and 75 % were ≥ 0.75 as compared to < 0.50 in 20 %, ≥ 0.50 to < 0.75 in 45 % and ≥ 0.75 in 35 % of parameters in the current study [32]. In the Arm position matching study, 25 % of parameters were ≥ 0.50 to < 0.75 and 75 % were ≥ 0.75 as compared to < 0.50 in 13 %, ≥ 0.50 to < 0.75 in 50 % and ≥ 0.75 in 27 % in the current study [27]. Both studies included individuals with broad functional levels ranging from normal healthy adults to those with significant neurological impairments associated with stroke. The current study included only normal healthy children and adolescents, none with sensorimotor impairment. We suspect that we observed lower ICCs in the present study as a direct result of failing to include individuals with sensorimotor impairments. Inclusion of such individuals in prior studies demonstrated that the robotic scores had relatively low intra-subject test-retest variability across a large range of possible values which led to moderate to high ICCs in the overwhelming majority of parameters. In the present study we observed low ICCs in parameters where we recorded a very small range of scores across subjects (e.g., Visually guided reaching, Initial Speed Ratio – 0.92 to 1.0). In the future we plan to re-evaluate the reliability of these parameters in children with brain injury.

Results from the current study are similar to those from a test-retest reliability study (tested 60 days apart) that evaluated a battery of neuropsychological tests, including the Trail making B task in children ages 9-14 [23]. As in the current study only healthy typically developing children with no neurological impairments were included in the reliability analysis, intra-class correlation coefficients ranged broadly from poor to good, 0.46 to 0.83 respectively [23]. The intra-class correlation coefficients for total time (s) associated with the Trail making B task in our study was 0.44 (S1 to S2) as compared to 0.65 in the previous study [23].

In the studies that included the KINARM robot tasks of Visually guided reaching and Arm position matching, data from both patients and controls were included in the intra-class correlation coefficients computation. The presence of sensorimotor impairment in a portion of the population included in these reliability studies resulted in an increased level of variability associated with the outcome measures [27, 33]. This was not the case in the current study or the paper that included the Trail making B task, individuals who had sustained a concussion were not included in the reliability testing [23]. Taken together these results suggest that the level of performance variability associated with the neurological impairment post-stroke appears far greater than that associated with neural development. This is an important distinction as variability among subjects’ scores must be large to demonstrate reliability [43]. Thus, we posit that the low intra-class correlation coefficients seen in the current study may have resulted from less variability among subjects due to the fact that all participants were healthy typically developing children with no neurological impairments. This is one limitation of the study. This is an argument for the inclusion of individuals across a broad functional spectrum when testing the reliability of any measurement tool.

When Bland-Altman plots were used to determine agreement with respect to the difference score associated with subject’s performance for S1 to S2 or S2 to S3 a learning effect became apparent in a few of the parameters in the current study. Figure 3 presents examples of two parameters that reflect agreement in performance from S1 to S2 and S2 to S3, whereas Fig. 4 shows examples of two parameters that reflect the presence of a learning effect for S1 to S2 but not S2 to S3. Many of the parameters that showed improvement in the Bland-Altman plots showed a statistically significant increase in performance from S1 to S2, which reflected a learning effect, but not S2 to S3 (refer to Table 5). In addition, improved performance was identified when S1 was compared to S3. This shows that the learning effect had stabilized by the third completion of the tasks. As seen in Fig. 4a the improvement in performance associated with the total hits (Object hit) resulted in a negative shift in the difference scores. However, improved performance associated with total time (s) (Trail making B) seen in Fig. 4c resulted in a positive shift in the difference scores. Thus dependent upon the nature of the skill being evaluated improvement in parameter performance was reflected either as an increase or decrease in value. When evaluated, clinically relevant changes were seen only in parameters from Object hit, Object hit and avoid, and Trail making B.

A significant improvement or learning effect has been seen with the use of the paper and pencil version of the Trail making B task in children, adolescents and adults [23, 47, 48]. Practice effects tend to be defined as some improvement in performance between concurrent test sessions based on familiarity with the procedures and/or previous exposure to the assessment, while learning effects relate to the retention of the improvement over a period of time [22, 49]. The results in our study showed that the learning effect had stabilized by the third application of the test.

Learning effects can be a confounding factor in the interpretation of test scores. The inherent variability associated with a learning effect may artificially inflate intra-class correlation coefficients. This is a limitation when using intra-class correlation coefficients to evaluate reliability and highlights the importance of using more than one method when testing reliability. We can speculate that some of the intra-class correlation coefficients in the current study may have fallen within the low to moderate range in the absence of a learning effect. The Bland-Altman plots however show good absolute reliability in the majority of the parameters for S1 to S2 and all parameters for S2 to S3, with stabilization of the learning effect.

If not addressed, learning effects have the potential to falsely give the impression of “improvement” which can adversely complicate interpretation of outcomes particularly when comparing athletes from pre-season performance to post-concussion. In one reliability study that evaluated neuro-cognitive tests, the learning effect was adjusted using a correction factor; a mean change score was calculated and added to the confidence interval of the outcome scores after repeat testing [23]. Under these conditions no change in performance could be interpreted as absence of neural impairment following concussion. An alternative interpretation would be that the robot tasks did not target areas of the brain susceptible to concussion or that the robot was not able to pick up any change in performance. A significant decline in performance could be attributed to the effect of the concussion. A correction factor was not calculated in the current study.

Since the effect of learning was stable after the second testing session an alternative strategy would be to implement a practice session (P1) preceding experimental testing in the pre-season [50, 51]. This would eliminate the effect of learning and changes in performance outcomes post-injury could be directly linked to the effects of concussion. Increased performance outcomes following concussion, with no practice session, could be interpreted as a lack of neural impairment. Alternatively, no change in performance could suggest the presence of neural impairment, as an increase in performance would be expected in the presence of learning. If a practice session (P1) were included in the experimental paradigm no change in performance from pre-season testing (S1) to post-concussion assessment (S2) would be interpreted as clinically insignificant, no neural impairment. Similarly, with no practice session, a significant increase from S1 to S2 would be interpreted as an absence of neurological impairment. In those parameters that did not show a clinically significant learning effect, no change in performance post-concussion would be interpreted as the absence of neural impairment whereas a decline in performance would be associated with the effect of concussion.

## Conclusion

In general, the relative homogeneity of the sample as well as the effect of learning seen in some of the outcome parameters appear to have had a negative impact on the intra-class correlation coefficients for session 1 to 2, with less impact for 2 to 3. However, the Bland-Altman analysis supports good absolute reliability. Thus the KINARM robot appears to be reliable for healthy male children with no neurological impairment ranging in age from 10 to 14. Our finding supports further testing to evaluate children pre and post-concussion, to determine whether the KINARM robot is sufficiently sensitive to identify neural impairments. The learning effect could be addressed in one of two approaches by: 1) creating a learning effect correction adjustment factor which would enhance efficiency at baseline testing and/or 2) implementing a training practice session to eliminate the learning effect. These findings begin to establish a group of parameters associated with KINARM robot tasks appropriate for the use in young sports participants and “set the stage” for clinical studies to evaluate the validity of these measures in a younger population.
